# Influenza vaccination in patients with heart failure: A protocol for meta-analysis of randomized controlled trials

**DOI:** 10.1097/MD.0000000000028844

**Published:** 2022-02-11

**Authors:** Hidekatsu Fukuta, Hiromi Hagiwara, Takeshi Kamiya

**Affiliations:** a Core Laboratory, Nagoya City University Graduate School of Medical Sciences, Nagoya, Japan; b Department of Medical Innovation, Nagoya City University Graduate School of Medical Sciences, Nagoya, Japan; c Department of Medical Innovation, Nagoya City University Graduate School of Medical Sciences, Nagoya, Japan.

**Keywords:** heart failure, influenza, meta-analysis, vaccine

## Abstract

**Background:**

Heart failure is a major public health problem. Although there have been significant advances in the management of heart failure, the mortality and morbidity in heart failure patients remain high. Heart failure patients are susceptible to influenza-related complications including acute heart failure exacerbations and secondary infections such as pneumonia, both of which lead to significant morbidity and mortality. An earlier meta-analysis of observational cohort studies reported that influenza vaccination was associated with reduced risk of mortality in heart failure patients. Although there are no published randomized controlled trials (RCTs) on the effect of influenza vaccination on clinical outcomes in heart failure patients, there are several on-going RCTs examining the effect in these patients. We aim to conduct a meta-analysis of RCTs to assess the efficacy and safety of influenza vaccination in heart failure patients.

**Methods:**

This meta-analysis will include RCTs examining the effect of influenza vaccination in heart failure patients. Information of studies will be collected from electronic databases. The primary outcome of interest will be cardiovascular death. The secondary outcomes of interest will be all-cause death, nonfatal myocardial infarction, nonfatal stroke, hospitalization for heart failure, and hospitalization for any cause.

**Discussion:**

This meta-analysis will evaluate the efficacy and safety of influenza vaccination in heart failure patients, providing evidence to the use of influenza vaccine in these patients.

**Systematic review registration:**

INPLASY202210115.

## 1 Introduction

Heart failure is a major public health problem, with a prevalence of more than 5.8 million in the United States and more than 23 million worldwide.^[1]^ Although there have been significant advances in the management of heart failure, the mortality in patients with heart failure remains high with 50% dying within 5 years.^[1]^ Heart failure patients are susceptible to influenza-related complications including acute heart failure exacerbations and secondary infections such as pneumonia, both of which lead to significant morbidity and mortality.^[2–4]^ The effect of influenza vaccination on clinical outcomes in heart failure patients has been reported in many observational cohort studies.^[5–10]^ An earlier meta-analysis of these observational studies reported that influenza vaccination was associated with reduced risk of mortality during 1-year and long-term follow-ups in heart failure patients.^[11]^ Although there are no published randomized controlled trials (RCTs) on the effect of influenza vaccination on clinical outcomes in heart failure patients, there are several on-going RCTs examining the effect in these patients.^[12]^ We aim to conduct a meta-analysis of RCTs to assess the efficacy and safety of influenza vaccination in heart failure patients.

## 2 Methods

This study has been registered on International Platform of Registered Systematic Review and Meta-analysis Protocols with registration number of INPLASY202210115 (https://www.doi.org; DOI: 10.37766/inplasy2022.1.0115). This protocol for meta-analysis will be performed according to the Preferred Reporting Items for Systematic Review and Meta-analysis Protocols (PRISMA-P) statement.^[13]^

### 2.1 Search strategy

The electronic databases for literature search will include PubMed, Scopus, Cochrane Library, and Web of Science. For search of the eligible studies, the following key words and Medical Subject Heading will be used: *heart failure, influenza, vaccine, randomized*. Only articles published in the English language will be included.

### 2.2 Study design

RCTs will be included for this meta-analysis. Observational studies will not be included.

### 2.3 Selection criteria

Studies will be considered eligible if they; included heart failure patients; were RCT; used influenza vaccine; and compared with usual medical therapy or placebo control group.

### 2.4 Outcomes

The primary outcome of interest will be cardiovascular death. The secondary outcomes of interest will be all-cause death, nonfatal myocardial infarction, nonfatal stroke, hospitalization for heart failure, and hospitalization for any cause.

### 2.5 Data extraction

Information on the study and patient characteristics, methodological quality, intervention strategies, and clinical outcomes will be systematically extracted separately by 2 reviewers. Disagreements will be resolved by consensus.

### 2.6 Quality assessment

The Cochrane Risk of Bias tool will be used to assess the quality of included RCTs.^[14]^ The quality of evidence for the outcomes will be evaluated by use of the Grading of Recommendations Assessment, Development and Evaluation (GRADE) system.^[15]^ The quality of evidence will be evaluated across the domains of risk of bias, consistency, directness, precision, and publication bias.

### 2.7 Statistical analysis

For each outcome, the pooled estimate of hazard ratio and 95% CI will be calculated with a fixed-effects model. The heterogeneity will be assessed using the Cochran Q chi-square test and *I*^2^ statistic; for the Cochran Q chi-square test and *I*^2^ statistic, a *P* value of <.1 and *I*^2^ > 50%, will be considered significant, respectively.^[16]^ When there is significant heterogeneity, the data will be pooled using a random-effects model. A two-tailed *P* < .05 will be considered statistically significant. Publication bias will be assessed graphically using a funnel plot and mathematically using Egger test.

### 2.8 Sensitivity analysis

Meta-regression will be used to determine whether the effect of influenza vaccination is confounded by baseline clinical characteristics. Meta-analysis will be performed separately for patients with reduced ejection fraction and those with preserved ejection fraction.

### 2.9 Ethical issues

This meta-analysis is a literature study. Ethical approval is not required because this meta-analysis will not involve any subject directly.

## 3 Discussion

To the best of our knowledge, this is the first meta-analysis of RCTs examining the effect of influenza vaccination on clinical outcomes in heart failure patients. Although the guidelines of Heart Failure Society of America, annual influenza vaccination is recommended in all heart failure patients in the absence of known contraindications, the recommendation is largely based on observational data and expert opinion.^[17]^ Our meta-analysis will provide a higher level of evidence for recommendation for the use of influenza vaccine in heart failure patients.

## Author contributions

All authors critically revised the manuscript.

**Conceptualization:** Hidekatsu Fukuta.

**Drafted manuscript:** Hidekatsu Fukuta.

**Literature retrieval:** Hidekatsu Fukuta, Hiromi Hagiwara.

**Methodology:** Hidekatsu Fukuta, Hiromi Hagiwara.

**Supervision:** Takeshi Kamiya.
